# An Enhanced MIBKA-CNN-BiLSTM Model for Fake Information Detection

**DOI:** 10.3390/biomimetics10090562

**Published:** 2025-08-23

**Authors:** Sining Zhu, Guangyu Mu, Jie Ma, Xiurong Li

**Affiliations:** 1 International Business School, Jilin International Studies University, Changchun 130117, China; zhusining@jisu.edu.cn; 2 School of Management Science and Information Engineering, Jilin University of Finance and Economics, Changchun 130117, China; 3 Office of Scientific Research, Jilin University of Finance and Economics, Changchun 130117, China; majie@jlufe.edu.cn; 4 Faculty of Information Technology, Beijing University of Technology, Beijing 100124, China; xiurong_li@bjut.edu.cn

**Keywords:** fake information detection, black kite optimization algorithm, dual-channel, hybrid model, social media platforms

## Abstract

The complexity of fake information and the inefficiency of parameter optimization in detection models present dual challenges for current detection technologies. Therefore, this paper proposes a hybrid detection model named MIBKA-CNN-BiLSTM, which significantly improves detection accuracy and efficiency through a triple-strategy enhancement of the Black Kite Optimization Algorithm (MIBKA) and an optimized dual-channel deep learning architecture. First, three improvements are introduced in the MIBKA. The population initialization process is restructured using circle chaotic mapping to enhance parameter space coverage. The conventional random perturbation is replaced by a random-to-elite differential mutation strategy (DE/rand-to-best/1) to balance global exploration and local exploitation. Moreover, a logarithmic spiral opposition-based learning (LSOBL) mechanism is integrated to dynamically explore the opposition solution space. Second, a CNN-BiLSTM dual-channel feature extraction network is constructed, with hyperparameters such as the number of convolutional kernels and LSTM units optimized by MIBKA to enable adaptive model structure alignment with task requirements. Finally, a high-quality fake information dataset is created based on social media platforms, including CCTV. The experimental results show that our model achieves the highest accuracy on the self-built dataset, which is 3.11% higher than the optimal hybrid model. Additionally, on the Weibo21 dataset, our model’s accuracy and F1-score increased by 1.52% and 1.71%, respectively, compared to the average values of all baseline models. These findings offer a practical and effective approach for detecting lightweight and robust false information.

## 1. Introduction

With the Internet’s widespread adoption and rapid development, social media platforms have become convenient spaces for users to access information, share opinions, and communicate daily [1,2]. Users have transformed from passive information consumers to active content creators and distributors, accelerating the rapid dissemination of fake information across social networks [3,4]. Fake information could be defined as intentionally or unintentionally disseminated content that violates authenticity, including false statements, manipulated facts, and misleading narratives, with the potential to cause societal harm [5,6]. In 2025, fake news continues to pollute the Internet at an alarming scale, with 62% of online content now deemed false. A staggering 86% of global citizens have been exposed to misinformation, while 40% of content shared on social media is fake [7]. False information spread by social media has amplified its disruptive effects, disturbed daily life, and posed serious challenges to public safety and governance. Therefore, exploring rapid and efficient methods for fake information detection has become a persistent research focus in academia.

Traditional fake information detection methods rely on manually defined linguistic features, such as sentiment polarity, lexical complexity, and propagation rule models [8,9]. However, these approaches struggle with significant limitations in generalization when confronted with adversarially generated fake texts. The advancement of pre-trained language models has introduced new characteristics in fake information, including logical closed loops and contextual adaptation [10]. These developments present dual challenges for detection technologies. Detection methods must effectively capture semantic contradictions within texts while modeling the evolving patterns during information dissemination [11,12]. Deep learning-based detection methods have emerged as a crucial technological approach to tackle these difficulties due to their powerful feature abstraction capabilities [13,14].

In the domain of fake information detection, the integration of Convolutional Neural Networks (CNN) and bidirectional long short-term memory networks (BiLSTM) has demonstrated unique value [15]. CNN accurately captures local semantic patterns through hierarchical convolution operations [16]. These operations effectively identify unconventional modifier stacking and abnormal anaphoric relations, which are micro-linguistic features essential for precise detection. BiLSTM models bidirectional temporal dependencies and effectively tracks the logical coherence of textual context [17]. As a result, they are particularly well-suited for analyzing the progressive semantic distortion that occurs during the propagation of fake information. Compared to commonly used Transformer architectures, this combination offers irreplaceable advantages in specific scenarios. In data-scarce contexts, such as detecting fake information during emerging events, Lightweight recurrent neural networks such as BiLSTM can compress the number of parameters to less than 1/50 of the Transformer model while maintaining a performance level of over 90% in text classification tasks [18]. Additionally, the inference speed of CNNs in short text classification tasks is 3 to 5 times faster than that of Transformer-based models, and the accuracy is comparable in scenarios with high-frequency vocabulary [19]. Nevertheless, the performance of such models heavily depends on hyperparameter configuration [20]. Traditional grid search methods may require thousands of GPU hours when optimizing complex parameters, resulting in substantial human and computational costs. Gradient-based optimization strategies struggle with discrete parameter combinations, leading to high tuning costs and poor cross-domain robustness in practical deployments [21].

Researchers are exploring swarm intelligence algorithms to improve efficiency in optimizing deep learning model parameters. From classical particle swarm optimization (PSO) and genetic algorithms (GA) to emerging Black Kite Algorithm (BKA), these methods simulate collective behaviors in biological populations and demonstrate unique advantages in tackling high-dimensional, non-convex optimization problems [22,23,24]. Taking BKA as an example, it simulates the circling foraging behavior of black kites by dividing population individuals into explorers and followers and dynamically adjusting their movement strategies during the search. Compared with traditional algorithms, BKA exhibits faster convergence in continuous parameter optimization tasks. However, finding optimal solutions can still be challenging. This difficulty largely stems from imbalances between global exploration and local search capabilities, as well as BKA’s limited global search capability and vulnerability to local optima. Therefore, it is necessary to enhance BKA.

Here, we can obtain the following two motivations for this study:

(1) The dual-channel integrates local and global semantic understanding, enabling comprehensive characterization of fake information’s diverse linguistic features.

(2) The improved BKA model can optimize the hyperparameters of the hybrid deep learning model, enabling it to achieve higher accuracy.

Thus, this study proposes an improved multi-strategy Black Kite Algorithm (MIBKA) and constructs the MIBKA-CNN-BiLSTM hybrid model. The innovations are reflected in the following aspects.

(1) The paper proposes three strategies to enhance the BKA. The circle chaotic mapping is used for population initialization to make the initial population distribution more uniform. A differential elite mutation strategy is integrated during the kite’s attack phase for position updates to balance global exploration and local exploitation. An opposition-based learning mechanism is introduced for an individual foraging to dynamically explore the op-position solution space.

(2) A dual-channel feature extraction network combining CNN and BiLSTM is proposed. The CNN branch employs multi-scale convolution kernels to capture local textual anomaly patterns, while the BiLSTM branch models contextual logical relations through hierarchical state propagation. Then, the improved MIBKA is used to optimize hyperparameters, including the number of 1D convolution filters, convolution kernel sizes, and the number of BiLSTM units.

(3) A fake information dataset is constructed and validated. Extensive comparative experiments with various single and hybrid deep learning models demonstrate the superiority of the MIBKA-CNN-BiLSTM model in fake information detection tasks.

## 2. Related Work

### 2.1. Traditional Techniques-Driven Fake Information Detection

Traditional fake information detection techniques focus on two main approaches, manual rule construction and shallow feature analysis, corresponding to two foundational research frameworks driven by expert knowledge and data statistics [25].

Rule-based methods depend on domain experts to systematically extract linguistic and logical features, thereby constructing detection systems [26]. The core lies in developing a rule base encompassing multidimensional linguistic anomaly patterns. Specifically, the rule-based methods can be categorized into three distinct types. The first aspect focuses on the syntactic level, identifying contradictory rhetorical patterns where absolutist statements coexist with unsupported claims [27,28]. Thus, rules are established that capture common linguistic exaggerations and subjective judgments to detect emotionally charged fake information. The second type addresses the logical level by identifying gaps in causal relations caused by insufficient links between premises and conclusions [29]. It extracts structured patterns that indicate broken reasoning chains, thereby improving the detection of argumentative fallacies in misleading content. The third point examines inconsistencies in time and events [30]. It identifies conflicts in the events’ timing and sequences by finding contradictory adverbial phrases. It also sets up rules to analyze event consistency to spot misinformation based on fake timelines. Verified through manually annotated samples, these rule sets demonstrate precise localization capabilities and strong interpretability in identifying typical linguistic anomalies. Although rule-based methods provide explainability advantages, their passive knowledge acquisition remains a fundamental limitation. As creators of fake information adopt more flexible strategies, experts must frequently review new fake texts and enhance their guidelines, increasing maintenance costs as the types of information grow.

Research has gradually shifted toward shallow feature analysis to overcome the shortcomings of rule-based methods in generalization and scalability. This approach utilizes machine learning techniques to convert text into quantifiable feature vectors, enabling automated detection [31,32]. Some researchers often prepare texts by breaking them down into smaller parts and analyzing the role of each word. They look at features such as word frequency, sentence complexity, and the overall tone of the text. After this analysis, they create models to detect patterns using tools like Support Vector Machines and Random Forests [33]. Other researchers have identified distinct patterns over time in the detection features that differentiate genuine information from fake information. They suggest using dynamic temporal structures to capture the evolution of these time-sensitive features. They employ machine learning algorithms such as decision trees, random forests, and support vector machines (SVM) to classify this information. The primary advantages of shallow feature analysis lie in relatively controllable modeling processes, low computational complexity, and high training efficiency with strong interpretability, particularly on medium-scale datasets [34,35]. Furthermore, the sensitivity of shallow models to feature weights provides clear discriminative criteria, which is beneficial in situations that require explicit explanations of detection logic. However, when false information is hidden through semantic operations like local data tampering and restructuring narrative logic, traditional feature engineering often fails to identify the deep logical contradictions within the text effectively. This limitation stems from over-reliance on handcrafted features and the inability to model nonlinear semantic relationships between linguistic units. Therefore, deep learning techniques offer promising new pathways to break traditional detection performance bottlenecks.

### 2.2. Deep Learning-Driven Fake Information Detection

Advances in deep learning have transformed fake information detection from basic, feature-based classification to deep semantic understanding through end-to-end neural architectures. This evolution has led to three major technical branches: single models, hybrid architectures, and pre-training enhanced models [36,37,38].

Initial deep learning explorations in fake information detection focus on constructing structurally clear and task-specific single models, including Convolutional Neural Networks (CNN), Recurrent Neural Networks (RNN), Long Short-Term Memory networks (LSTM), Gated Recurrent Units (GRU), and early Transformer variants [39,40,41,42]. CNNs excel at extracting local syntactic and phrase-level features, while RNNs capture temporal dependencies to identify semantic continuity anomalies in context. LSTMs mitigate the vanishing gradient problem in long-distance dependencies via forget and memory gates, showing a strong ability to maintain semantic coherence. GRUs simplify gating mechanisms further, achieving faster convergence and comparable performance in small-data scenarios. Transformers possess specific global attention capabilities suitable for loosely structured social texts. Due to their structural simplicity and controllable parameterization, single-model architectures offer advantages such as stable training, fast convergence, and ease of debugging. These characteristics make them well-suited for early-stage scenarios with limited sample size or when interpretability is a primary concern. In addition, such models still demonstrate a strong ability to detect specific types of fake content, particularly in patterned rumors or highly repetitive texts. Nevertheless, single models typically concentrate on a limited range of semantic features due to their simplistic structure. They cannot capture the diverse manifestations of misinformation that may arise locally and globally across multiple granularities and contexts. Consequently, they struggle to meet the demands of comprehensive, multi-perspective modeling of high-dimensional deceptive patterns.

To overcome the dimensional limitations of single models, researchers propose various hybrid neural network structures that integrate different model advantages, enhancing the modeling capacity for complex semantics of fake information. Among these hybrid models, local-global fusion architectures are most prevalent. CNN-RNN or CNN-BiLSTM synergistically models local phrase features and global temporal dependencies [43,44]. CNN layers efficiently identify lexical-level anomalies, while BiLSTM captures inter-sentence logical relations and long-term semantic evolution, improving contextual sensitivity and robustness against complex fake information. Additionally, attention mechanisms have also been incorporated into hybrid models. For instance, the CNN-BiGRU-Attention model focuses on key semantic regions post-local feature extraction, improving recognition of covert manipulative language [45]. Some studies have explored the combination of Graph Neural Networks (GNN) with sequential models by constructing user-content interaction graphs to extract propagation structure features and model the semantic evolution in dissemination [46,47]. This approach enhances the depiction of fake information spread patterns and content dynamics. The advantages and limitations of the above models are shown in Table 1. In summary, hybrid architectures integrate multi-level semantic information deeply while ensuring computational efficiency. They significantly enhance the detection of logical inconsistencies, emotional polarization, and false citations, establishing themselves as valuable methods in fake information detection.

With the rise in pre-trained language models like Bidirectional Encoder Representation from Transformers (BERT), researchers have used BERT to analyze contextual semantics and develop deep semantic vector representations. BERT successfully captures long-range dependencies and implicit semantic connections by implementing self-attention mechanisms [48,49]. Improving the model enhances its comprehension of intricate text structures and demonstrates robustness in detecting disguised and semantically ambiguous misinformation. Some studies integrate BERT with BiLSTM and similar architectures to enhance modeling effectiveness [50]. These improvements result in hybrid networks that combine contextual modeling with temporal semantic mechanisms, thereby increasing detection accuracy and generalization capabilities. However, it is noteworthy that such large-scale language models still face significant challenges in practical deployment. Their massive parameter counts result in high computational costs, requiring powerful GPU clusters or cloud resources [51,52]. Additionally, adjustments in hyperparameters like the number of convolutional kernels, the depth of recurrent layers, and the number of attention heads can lead to significant performance variations. Therefore, creating efficient hyperparameter optimization methods for consistent performance improvements continues to be a key area of research.

## 3. Methodology

**Figure 1:**
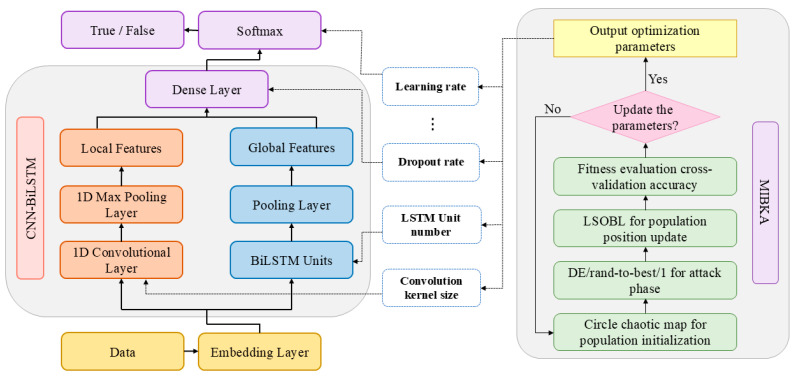
Architecture of the proposed MIBKA-CNN-BiLSTM model.

The paper proposes the MIBKA-CNN-BiLSTM hybrid model for fake information detection. First, the original BKA is enhanced using three strategies. Then, the MIBKA is employed to optimize the hyperparameters of the CNN-BiLSTM model. Finally, the trained MIBKA-CNN-BiLSTM model is used to classify the crawled information as real or fake. The overall architecture of the proposed model is illustrated in Figure 1.

### 3.1. Black Kite Optimization Algorithm (BKA)

The black kite is a medium-sized bird of prey known for its exceptional agility in hovering, strategic hunting maneuvers, and adaptive migratory patterns. These traits enable it to explore and exploit resources in diverse environments efficiently. The Black Kite Optimization Algorithm (BKA) is a nature-inspired optimization method derived from these distinctive behaviors. The BKA consists of three main stages: population initialization, attack behavior, and migratory behavior.

#### 3.1.1. Population Initialization

In BKA, the first step is to create a set of random solutions to initialize the population. The following matrix can be used to represent the position of each Black Kite (BK).

(1) $$BK=\left[\begin{array}{cccc} BK_{1,1} & BK_{1,2} & \ldots & BK_{1,dim}\\ BK_{2,1} & BK_{2,2} & \ldots & BK_{2,dim}\\ \vdots & \vdots & \vdots & \vdots \\ BK_{pop,1} & BK_{pop,2} & \ldots & BK_{pop,dim} \end{array}\right]$$

Here, pop denotes the population size, and dim refers to the dimensionality of the search space. ${BK}_{i,j}$ represents the position of the i black kite in the j dimension. The positions are initialized according to Equation (2).

(2) $${BK}_{i,j}=BK_{lb}+\mathrm{rand}\left(BK_{ub}-BK_{lb}\right)$$

i is an integer between 1 and pop. $BK_{ub}$ and $BK_{lb}$ represent the lower and upper bounds of the j dimension, and rand is a randomly generated number within the range [0, 1]. During the initialization phase, the individual with the best fitness is selected as the leader of the initial population, as shown in Equation (3).

(3) $$f_{best}=\min\left(f{(X}_{i})\right)$$

#### 3.1.2. Attack Behavior

Black kites, while flying, adjust the angles of their wings and tails based on the wind speed. This flexibility allows them to hover silently and observe their prey before diving quickly to attack. The attack behavior is modeled using global exploration and local exploitation. The position update rule is formulated in Equation (4).

(4) $$X_{t+1}^{i,j}=\left\{\begin{array}{c} X_{t}^{i,j}+n\left(1+sin(r\right))X_{t}^{i_{,}j}\;\;\;p<r\\ X_{t}^{i,j}+n\left(2r-1\right)X_{t}^{i,j}\;\;\;\;\;\;\;\;\;\;\;\;\;else\; \end{array}\right.$$

The first case simulates scenarios where the black kite hovers in the air and then dives rapidly toward its prey. The second case simulates hovering and gradual movement. $X_{t+1}^{i,j}$ and $X_{t}^{i,j}$ denote the j dimension position of the i black kite at iterations t and t+1. r∈ [0, 1] is a random number, and p is a constant set to 0.9. The parameter n is a nonlinear control variable, defined in Equation (5), where T is the maximum number of iterations, and t is the current iteration number. This parameter is designed to adjust the search scope dynamically.

(5) $$n=0.05\times \exp\left(-2\times {\left(t/T\right)}^{2}\right)$$

#### 3.1.3. Migratory Behavior

In nature, black kites migrate to new habitats for better survival conditions and resources. When the current leader fails to find optimal environments, the group dynamically replaces it with more capable individuals, ensuring continuous movement toward better habitats. This process is similar to the dissemination process of false information, where key communicators guide the spread of the information. Therefore, the migration behavior of the black kites can inspire the identification of the adaptive transmission patterns of false information. Migration is typically led by a dominant bird whose navigation ability is critical for group success. BKA adopts a migratory strategy based on the assumption that if the fitness of the current individual is worse than that of a randomly selected individual, the leader should relinquish control and join the migratory group. Otherwise, the leader continues to guide the population toward the optimal solution. The migration-based position update rule is shown in Equation (6).

(6) $$X_{t+1}^{i,j}=\left\{\begin{array}{c} X_{t}^{i,j}+C\left(0,1\right)\times \left(X_{t}^{i,j}-L_{t}^{j}\right)\;\;\;\;\;\;\;\;\;\;\;\;\;\;F_{i}<F_{ri}\\ X_{t}^{i,j}+C\left(0,1\right)\times \left(L_{t}^{j}-m\times X_{t}^{i,j}\right)\;\;\;\;\;else\;\;\;\;\;\;\; \end{array}\right.$$

(7) $$m=2\times \sin\left(r+\pi /2\right)$$

Here, $L_{t}^{j}$ denotes the j dimension of the leader’s position at iteration t (i.e., the current global best solution). $F_{i}$ is the fitness value of the current individual, and $F_{ri}$ is the fitness value of a randomly selected individual. $C\left(0,1\right)$ refers to a value sampled from the Cauchy distribution. The probability density function of the Cauchy distribution is defined in Equation (8). When δ = 1 and μ = 0, the distribution simplifies to its standard form, as shown in Equation (9).

(8) $$f\left(x,\delta ,\mu \right)=\frac{1}{\pi }\frac{\delta }{\delta^{2}+{\left(x-\mu \right)}^{2}},\;-\infty <x<\infty$$

(9) $$f\left(x,\delta ,\mu \right)=\frac{1}{\pi }\frac{1}{x^{2}+1},\;-\infty <x<\infty$$

### 3.2. Multi-Strategy Improved Black Kite Optimization Algorithm (MIBKA)

Although BKA demonstrates fast convergence speed and high optimization precision, global exploration and local exploitation are imbalanced. Specifically, its global search capability is relatively weak, making it prone to falling into local optima. Three improvement strategies are adopted in this study to address this issue.

#### 3.2.1. Introducing Circle Chaotic Mapping for Population Initialization

In swarm intelligence algorithms, the quality of the initial solution significantly impacts performance and convergence. The standard BKA utilizes a uniform random initialization strategy that may lead to uneven distribution and boundary aggregation in mixed-parameter spaces, thereby diminishing the effectiveness of global search. Alternative methods such as Latin hypercube sampling, low-discrepancy sequences like Halton, and chaotic mappings are commonly used to mitigate this issue. Chaotic mappings are preferred due to their ergodicity, randomness, and adaptability to complex parameter spaces.

This study evaluates 21 chaotic mappings, categorized into three types based on their dynamic characteristics, as summarized in Table 2.

**Figure 2:**
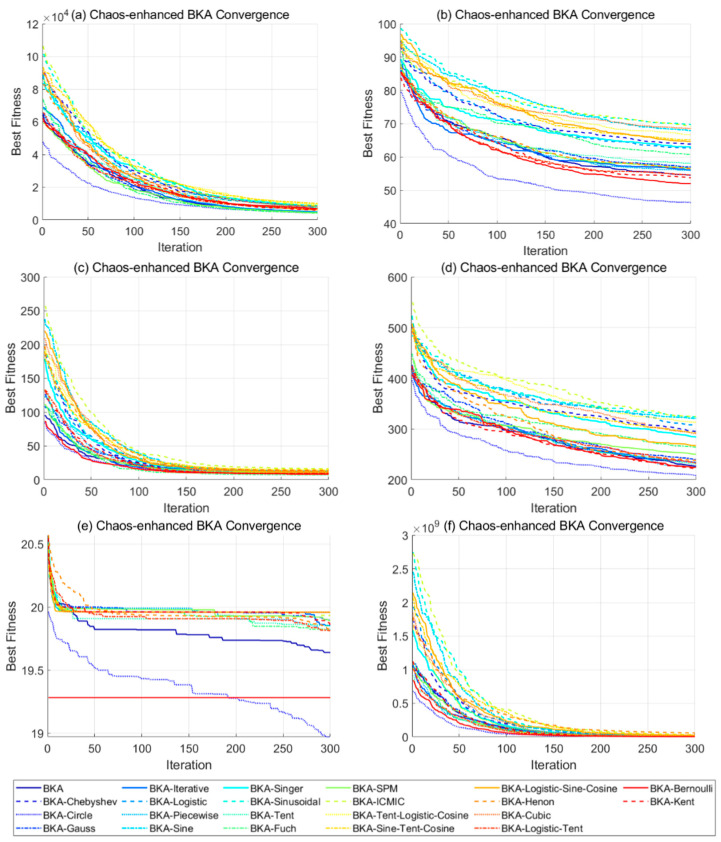
Best Fitness of BKA improved by different chaotic maps.

To determine the most suitable chaotic map for BKA initialization, each of the 21 strategies is tested by integrating it into BKA and evaluating the performance on the CEC2017 benchmark suite. The experiments are conducted with a population size of 50, a dimensionality of 30, and a maximum of 300 iterations. Each algorithm variant is run five times, and the average results are used for ranking. Evaluation metrics include optimal fitness, convergence iterations, and global optimality hit rate. Selected results are shown in Figure 2.

The experimental results show that the circle chaotic map outperforms other mappings on most benchmark functions. For instance, in the F1 test function, BKA with circle mapping achieves an average fitness of 8.3388 × 10^2^, reducing the original BKA result of 1.1078 × 10^3^ by 24.77%. The number of convergence iterations has decreased from 213 to 127, reflecting a 40.38% improvement in performance. Moreover, circle mapping ranks first in 21 out of 30 runs and exhibits premature convergence in only two cases. Based on these results, circle chaotic mapping is selected to improve the initialization phase of BKA.

The circle map generates pseudo-random sequences using a phase-shifting mechanism. Its iterative equation is defined in Equation (10). Here, θ = 0.5 is the rotation angle controlling the global traversal direction, and δ = 0.2 is the perturbation strength regulating local randomness.

(10) $$x_{k+1}=\left(x_{k}+\theta -\frac{\delta }{2\pi }\sin\left(2\pi x_{k}\right)\right)\mathrm{mod}-1$$

The improved initialization process is described as follows.

1. Chaotic sequence generation. For each individual, a chaotic sequence $\left\{x_{1},x_{2},\ldots ,x_{D}\right\}$ is generated from a random seed $x_{0}\;\epsilon$ [0, 1). The sequence is preheated for 100 iterations to eliminate transient effects. Here, D represents the number of parameters to be optimized.
2. Handling discrete parameters. For integer parameters such as the number of network layers $L\;\epsilon \left\{1,2,\ldots ,10\right\}$ or the number of convolution kernels $K\;\epsilon \left\{16,32,64\right\}$, floor mapping is applied to convert chaotic values. Specifically, given a normalized chaotic value $x_{chaos}$, the transformation is defined in Equation (11). $L_{max}$ and $L_{min}$ are the upper and lower bounds of the parameters. For example, if $x_{chaos}=0.73$, the convolution kernels K = $\left\lfloor 0.73\cdot \left(64-16\right)\right\rfloor$ + 16 = 51 is rounded and adjusted to 64.
  (11) $$L=\left\lfloor x_{chaos}\cdot \left(L_{max}-L_{min}\right)\right\rfloor +L_{min}$$
3. Preserving continuous parameters. For continuous variables such as learning rate α ϵ [0.0001, 0.1] and dropout rate β ϵ [0.2, 0.6], the original distribution of the chaotic sequence is retained through linear scaling, as shown in Equation (12). For instance, when $x_{chaos}$ = 0.15, then α = 0.0151.
  (12) $$\alpha =x_{chaos}\cdot \left(\alpha_{max}-\alpha_{min}\right)+\alpha_{min}$$
4. Dynamic boundary reflection. If $x_{k+1}$ exceeds the defined domain boundaries, a reflection mechanism is used to keep it within the feasible range, as defined in Equation (13). This strategy ensures valid solutions and maintains population diversity in the adequate search space.
  (13) $$x^{\prime }=\left\{\begin{array}{c} 2\cdot UB-x_{k+1},\;ifx_{k+1}>UB\\ 2\cdot LB-x_{k+1},\;ifx_{k+1}<LB \end{array}\right.$$

The BKA achieves better coverage and faster convergence in mixed parameter spaces by reconstructing the initialization phase using the circle chaotic mapping.

#### 3.2.2. Using Random-to-Elite Differential Mutation for Attack Phase

The core search mechanism of BKA differentiates roles between explorers and followers during the attack phase. Explorers expand the search space through stochastic movements, while followers focus on local exploitation around the current best solution. However, in complex high-dimensional mixed-parameter spaces, the original random perturbation strategy lacks guidance, challenging balancing global exploration and local exploitation. Some researchers have attempted to tune BKA’s internal parameters to address this limitation or integrate it with other metaheuristic algorithms. Although such methods can improve performance, they often face difficulties in parameter coordination. Compared with other approaches, mutation strategies introduce random or probabilistic perturbations to break population homogeneity. These strategies can be designed according to the specific problem and the characteristics of the algorithm. By incorporating historical information or elite individuals, they can guide the search direction more effectively. This approach helps achieve a better balance between global search and local exploitation but only requires a linear complexity of time.

This study investigates 13 mutation strategies, including Gaussian mutation (Guass1), elite Gaussian mutation (Guass2), Cauchy mutation (Cauchy1), inverse Cauchy mutation (Cauchy2), t-distribution mutation (t), adaptive t-distribution mutation (Self-t), normal cloud mutation (Cloud), periodic mutation (Periodic), elite differential mutation (DE/best/1), random-to-elite differential mutation (DE/rand-to-best/1), random differential mutation (DE/rand/2), best/2 mutation (DE/best/2), and non-uniform mutation (H). These strategies are categorized into four types based on their mathematical mechanisms, as shown in Table 3.

**Figure 3:**
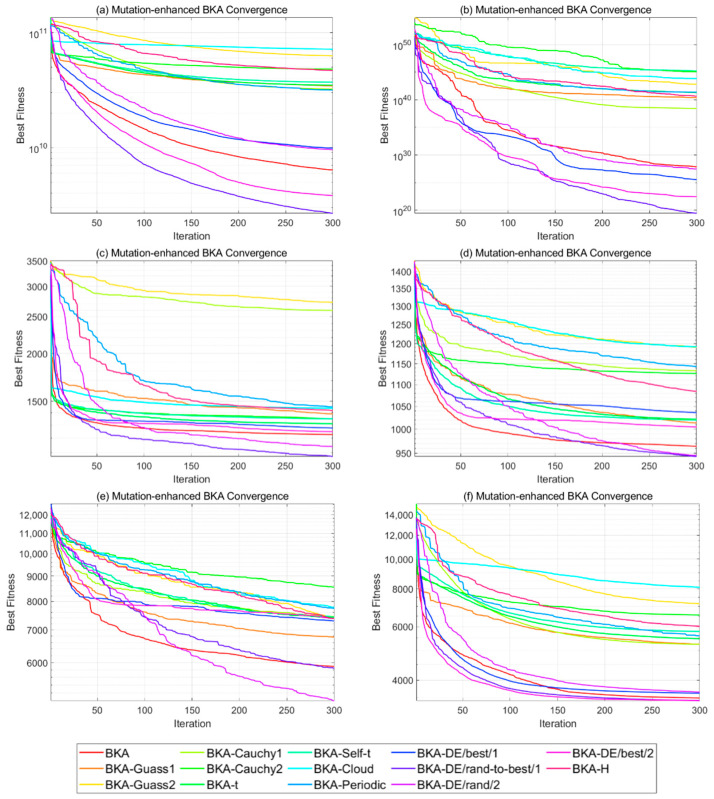
Best Fitness of BKA improved by different mutation strategies.

To identify the most effective strategy for improving BKA’s attack phase, each of the 13 mutation strategies is embedded into BKA and tested on the CEC2017 benchmark functions. Experiments are conducted with a population size of 50, a dimensionality of 30, and a maximum of 300 iterations. Every improved algorithm is executed five times, and average performance is recorded. Evaluation metrics included best fitness and number of convergence iterations. Selected results are illustrated in Figure 3.

Experimental results show that the random-to-elite differential mutation (DE/rand-to-best/1) performs best on 19 test functions. For instance, in the F10 benchmark test, the average fitness of this strategy reached 1.9581 × 10^9^, which was 80.42% lower than the original BKA’s 1.0002 × 10^10^. Differential evolution-based strategies balance exploration and exploitation by combining random exploration with elite guidance. Due to its random perturbation and elite direction characteristic, DE/rand-to-best/1 is integrated into BKA’s attack phase to compensate for the lack of guided search in the original design.

The random-to-elite differential mutation originates from the Differential Evolution (DE) algorithm, a population-based global optimization method. DE generates mutation vectors by computing individuals’ differences and combining them with crossover and selection to search for the global optimum. The DE/rand-to-best/1 strategy builds on this by introducing randomness to enhance diversity while leveraging elite individuals to accelerate convergence toward the global optimum.

In the BKA framework, this mutation strategy is embedded into the attack phase to replace the original stochastic disturbance formula. The updated position equation is defined in Equation (14).

(14) $$X_{t+1}^{i,j}=\left\{\begin{array}{c} X_{t}^{i,j}+n\left(1+sin(r\right))X^{i},\;j_{t}+F*\left(X_{t}^{r1,j}-X_{t}^{r2,j}\right)+\lambda *(X_{t}^{elite,j}-X_{t}^{i,j})\;\;\;p<r\\ X_{t}^{i,j}+n\left(2r-1\right)X_{t}^{i,j}+F*\left(X_{t}^{r1,j}-X_{t}^{r2,j}\right)+\lambda *(X_{t}^{elite,j}-X_{t}^{i,j})\;\;\;\;\;\;\;\;\;\;\;else\; \end{array}\right.$$

Here, $X_{t}^{r1,j}$ and $X_{t}^{r2,j}$ represent two randomly selected individuals from the population. $X_{t}^{elite,j}$ refers to the elite individual (with the best fitness) in the j dimension at iteration t. F is the differential scaling factor controlling the impact of the difference vector $\left(X_{t}^{r1,j}-X_{t}^{r2,j}\right)$. λ is the elite learning factor, which determines how much influence the elite solution exerts on the updated position.

By incorporating the DE/rand-to-best/1 strategy into BKA’s attack phase, the algorithm significantly reduces its susceptibility to local convergence. The enhanced equation combines random perturbation with elite-driven guidance, improving search efficiency and robustness across complex optimization tasks.

#### 3.2.3. Utilizing Logarithmic Spiral Opposition Learning for Position Update Phase

After completing the attack and migration phases, BKA only retains the superior individuals through fitness comparisons and lacks active exploration of the potential reverse solution space, resulting in a limited search range. In high-dimensional mixed parameter spaces, especially when discrete network layers coexist with continuous learning rates, forward search is prone to miss high-quality solutions in the reverse regions. Opposition-based learning (OBL) has been widely adopted among various improvement techniques due to its simplicity and effectiveness. The core idea of OBL is to generate opposite solutions relative to the current candidates and explore whether better solutions may lie in the opposite direction. This approach expands the search space, helps the algorithm avoid premature convergence, and enhances global optimization capability without significantly modifying the original algorithm structure. This study evaluates 12 opposition-based learning strategies and classifies them into five categories based on their core mechanisms, as shown in Table 4.

**Figure 4:**
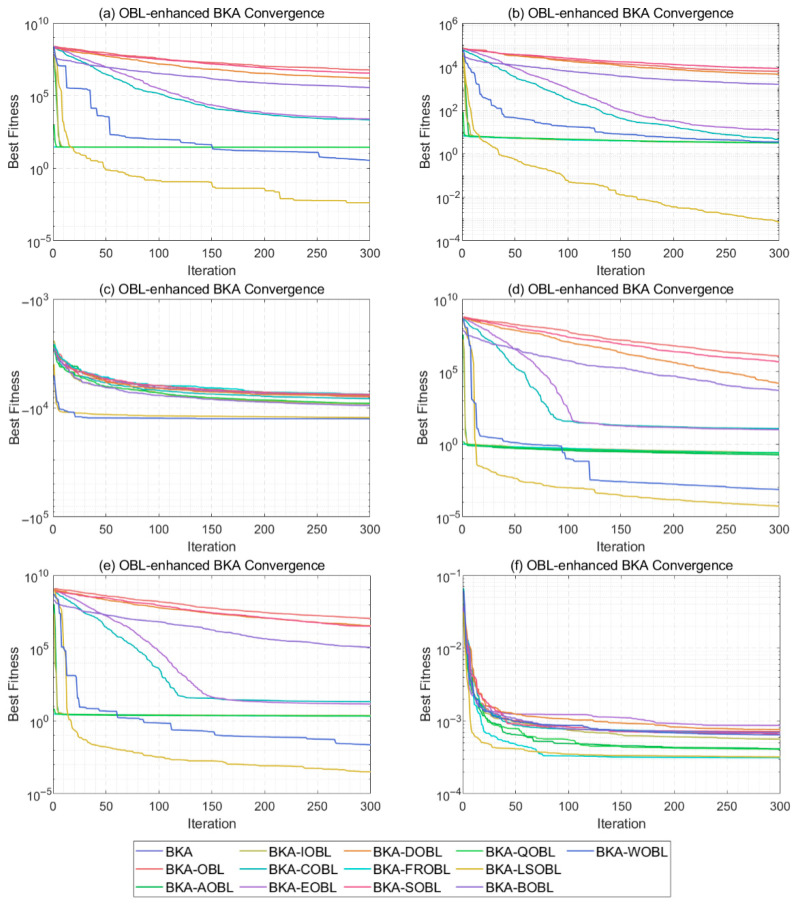
Best Fitness of BKA improved by different OBL strategies.

To identify the most suitable opposition learning strategy for enhancing the BKA population update phase, 12 strategies are individually integrated into BKA and tested on the CEC2017 benchmark set. Experiments are configured with a population size of 50, 30-dimensional search space, and 300 maximum iterations. Each modified algorithm is executed five times, and the average performance is recorded. Evaluation metrics include best fitness and number of convergence iterations. Partial results are illustrated in Figure 4.

Experimental results indicate that logarithmic spiral opposition-based learning (LSOBL) performs best on 24 benchmark functions. For the F23 function, the BKA-LSOBL algorithm converges in 102 iterations, while the original BKA takes 225 iterations, resulting in a 54.67% improvement. Its average fitness decreased from 1.204 × 10^4^ to 5.824 × 10^3^, representing a 51.63% reduction. Overall, LSOBL demonstrates the most significant enhancement among all tested strategies.

LSOBL is inspired by observing logarithmic spiral patterns in nature, which exhibit self-similarity and infinite scalability. These properties allow the algorithm to search effectively at multiple scales. In the context of opposition learning, the LSOBL strategy introduces a mathematical model based on the logarithmic spiral to generate opposite solutions. Compared to conventional OBL strategies, LSOBL enables wider yet directed exploration, accelerating convergence to the global optimum.

In the original BKA, the update mechanism retains the fitter individuals without exploring the solution space in the opposite direction. LSOBL introduces a spiral-based and dynamically bounded opposition learning model, mathematically defined in Equation (15).

(15) $$x_{spiral}=x_{best}\cdot {\mathrm{e}}^{k\theta }\cdot \cos\left(\theta \right)+\left(a_{t}+b_{t}-x\right)\cdot \left(1-{\mathrm{e}}^{-k\theta }\right)$$

Here, $x_{best}$ denotes the best individual in the current population. $\theta =2\pi \cdot t/T_{max}$ is the spiral angle that linearly increases with iteration. k = 0.2 controls the spiral’s tightness. $a_{t}=\mu_{t}-\delta_{t}$ and $a_{t}=\mu_{t}+\delta_{t}$ define the dynamic opposition boundaries. $\delta_{t}=\delta_{0}\cdot e^{-\lambda t}$ is a decay function that gradually reduces the boundary range.

This strategy is embedded into the population update phase of BKA and consists of three steps. (1) Generating opposite solutions based on a logarithmic spiral. (2) Discretizing and applying boundary truncation for discrete parameters. (3) When conducting a competitive update, only the better individual, either the original or the spiral-opposite candidate, is retained. The spiral mechanism dynamically balances global and local search. In the early stages, θ is small, and the spiral radius is large, promoting global exploration. In the later stages, as θ close to 2π, the spiral becomes tighter, focusing on local exploitation. The elite-guided term $x_{best}$ biases the search toward high-quality regions, avoiding ineffective exploration. A reflection boundary handling mechanism is applied when solutions exceed the defined domain for continuous parameters.

Incorporating LSOBL into BKA’s update mechanism significantly improves the algorithm’s global search ability and robustness. This enhancement effectively mitigates the limitations of the original BKA in dealing with unexplored solution spaces and premature convergence.

#### 3.2.4. Time Complexity Analysis of MIBKA

Time complexity is a critical metric for evaluating the efficiency of an algorithm, representing its performance in the worst-case scenario concerning input size. The core procedure of the MIBKA includes four major phases: chaotic initialization, attack, migration, and population update. Let N denote the population size, D the dimensionality of the search space, and $T_{max}$ the maximum number of iterations.

The time complexity of MIBKA is jointly determined by the baseline operations of the BKA and the additional overhead from the proposed improvement strategies. MIBKA employs the circle chaotic map to generate the initial population during the chaotic initialization phase. For each individual, 100 warm-up iterations are conducted to eliminate transient effects. Therefore, the initialization phase has a time complexity of $O\left(100\times N\times D\right)$, simplifying to $O\left(N\times D\right)$ as the constant factor is negligible in asymptotic analysis. During the attack phase, mutation operations inspired by differential evolution, combined with boundary reflection handling and discrete parameter rounding, are applied to each individual across all dimensions, resulting in a time complexity of $O\left(N\times D\right)$. The updates to positions during migration require traversing all individuals and computing dimensional updates, resulting in another $O\left(N\times D\right)$ complexity. The phase of population update involves generating logarithmic spiral-based opposition solutions, conducting validation filtering, and comparing elite steps, all of which contribute to the complexity of $O\left(N\times D\right)$. Fitness comparison requires $O\left(N\right)$, which is asymptotically dominated by the higher-dimensional operations. Thus, the overall complexity remains $O\left(N\times D\right)$.

In summary, the total time complexity per iteration of the MIBKA is $O\left(N\times D\right)$. Considering the entire optimization process over $T_{max}$ iterations, the overall time complexity becomes $O\left(T_{max}\times N\times D\right)$. Although MIBKA introduces additional procedures such as chaotic mapping, differential mutation, and opposition learning, these improvements only marginally increase the per-iteration computation. The algorithm retains the same polynomial-level complexity as the original BKA. Thus, MIBKA maintains strong scalability and practical efficiency for large-scale optimization problems.

#### 3.2.5. The Steps of MIBKA

MIBKA initializes the search space using the circle chaotic map, which ensures a uniformly distributed population. It employs an elite differential mutation strategy to balance global exploration and local exploitation during the optimization process. Furthermore, it integrates logarithmic spiral-based opposition learning to extend the search boundaries and avoid premature convergence. Throughout the optimization process, the model continuously evaluates the fitness of current candidate solutions, updates the direction of search based on elite individuals, and gradually converges towards the near-optimal solution. The complete pseudocode of MIBKA is presented in Algorithm 1.
**Algorithm 1: MIBKA (Multi-Strategy Improved Black Kite Algorithm)****Input:** Population size N, Dimensionality D, Max iterations T_max**Output:** Best solution x_best and its fitness f_best1:  Initialize population X = {x_1_, x_2_, ..., x_N} using circle chaotic map2:  Evaluate fitness f(x_i_) for each x_i_ ∈ X3:  Identify initial leader x_best with best fitness f_best4:  for t = 1 to T_max do5:   for each individual x_i_ ∈ X do6:    Generate rand ∈ [0, 1]7:    if rand < p_global then8:     // Global exploration9:     Update x_i_ using leader-based exploration rule10:   else11:    // Local exploitation12:    Update x_i_ near x_best using local refinement rule13:   end if14:   Apply random-elite differential mutation to x_i_15:   Evaluate new fitness f(x_i_)16:   if f(x_i_) better than f_best then17:    Update x_best ← x_i_, f_best ← f(x_i_)18:   end if19:  end for20:  // Migration phase21:  Generate new candidate x_new22:  Evaluate f(x_new)23:  if f(x_new) better than f_best then24:   Update x_best ← x_new, f_best ← f(x_new)25:  end if26:  // Spiral opposition-based population updateInput: Population size N, Dimensionality D, Max iterations T_max27:  for each x_i_ ∈ X do28:   Generate spiral-opposition solution x_i__opp29:   Evaluate f(x_i__opp)30:   if f(x_i__opp) better than f(x_i_) then

### 3.3. CNN-BiLSTM Model

To effectively capture local patterns and global semantic structures from textual data, we construct a dual-branch CNN-BiLSTM hybrid feature extraction model. This model extracts local linguistic features using a Convolutional Neural Network (CNN). At the same time, a bidirectional long short-term memory (BiLSTM) network is employed to model sequential semantic dependencies. The outputs of these two branches are concatenated and used for final classification.

The input text is first preprocessed and converted into a sequence of word embeddings with dimensionality d. Given an input text length of L, the embedded representation can be formulated as a matrix.

(16) $$X\in R^{L\times d}$$

Multiple one-dimensional convolutional kernels with varying sizes in the CNN branch are applied to capture multi-scale n-gram features. The feature map generated by the i convolutional kernel is computed as

(17) $$C_{i}=ReLU\left(W_{i}*X+b_{i}\right)$$

$W_{i}$ and $b_{i}$ denote the weight and bias of the i convolutional filter, respectively, and ∗ represents the one-dimensional convolution operation. ReLU is the activation function applied elementwise. All convolutional outputs are subject to max pooling along the temporal dimension and concatenated to form a fixed-dimensional local representation vector.

In the BiLSTM branch, the same input matrix X is fed into a bidirectional LSTM network to learn temporal dependencies. Let h denote the number of hidden units in each direction. The hidden state at time step t is computed as

(18) $$H_{t}=\left[\vec{h_{t}}\|\overset{\leftarrow }{h_{t}}\right]$$

The bidirectional hidden states are aggregated via average pooling or attention mechanisms to obtain a global semantic representation vector.

The outputs from the CNN and BiLSTM branches are concatenated to form the final text representation Z.

(19) $$Z=\left[{CNN}_{out}\|{BiLSTM}_{out}\right]$$

Then, the representation vector Z is passed through a fully connected layer, followed by a softmax to generate the predicted label.

The CNN-BiLSTM model involves several structural hyperparameters that significantly affect its performance, including the kernel sizes and counts in CNN, the number of LSTM layers and hidden units, the dropout rate, and the learning rate. These hyperparameters form a high-dimensional mixed search space, and their optimal configuration is crucial for the model’s predictive capability.

### 3.4. MIBKA-CNN-BiLSTM Model

To enhance the adaptability and parameter tuning efficiency of the CNN-BiLSTM model, we introduce a multi-strategy improved Black Kite Algorithm (MIBKA) to optimize the model’s hyperparameters jointly.

In this framework, the optimized hyperparameters include the number of convolutional kernels, the number of hidden units in the BiLSTM layer, learning rate, batch size, dropout rate, and additional parameters. MIBKA considers cross-validation accuracy to be the fitness function. During the initialization phase, the circle chaotic map is employed to enhance the diversity of initial solutions. A random-to-best differential mutation strategy is utilized during the optimization process to enhance local perturbation capabilities. An elite-driven updating mechanism is implemented to steer the population towards promising areas in the solution space. Moreover, logarithmic spiral-based opposition learning is embedded in the position update phase to improve the quality of solutions further. During each iteration, MIBKA uses the hyperparameter configuration represented by each individual to train a CNN-BiLSTM model and evaluates its performance on a validation set. Through continuous iteration, the algorithm converges toward a near-optimal set of hyperparameters. The final output of MIBKA is used to construct the optimized CNN-BiLSTM model, which is then evaluated on the test set.

This optimization framework enables the model to achieve improved training efficiency and predictive performance highlighting the practical value of MIBKA in complex, high-dimensional optimization scenarios.

## 4. Experiments

### 4.1. Experimental Setup

All experiments use an NVIDIA RTX 4090 GPU with 24 GB of VRAM in the MATLAB 2024b environment. The CNN-BiLSTM hybrid model is implemented using the Deep Learning Toolbox, and the optimization process is enhanced using the Parallel Computing Toolbox. The specific parameter settings are detailed in Table 5.

### 4.2. Evaluation Metrics

To assess the effectiveness of the proposed model, we select accuracy, precision, recall, and F1-score as metrics. The formulas for these metrics are shown in Equations (20)–(23). Table 6 uses TR and TF to denote True Real and True Fake, identifying the model and correctly predicting real and fake examples. Conversely, FR and FF represent False Real and False Fake, indicating that the model incorrectly predicts real and fake examples.

(20) $$Accuracy=\frac{TR+TF}{TR+FR+TF+FF}$$

(21) $$Precision=\frac{TR}{TR+FR}$$

(22) $$Recall=\frac{TR}{TR+FF}$$

(23) $$F1-score=\frac{2*Precision*Recall}{Precision+Recall}$$

### 4.3. Data Collection and Preprocessing

This study collects data from CCTV.com and various fact-checking platforms using web scraping technology to validate the model’s effectiveness. The dataset comprises verified real information published on CCTV.com between April 2023 and November 2024. After removing irrelevant or meaningless content, we obtain 2817 real information. Fake information comes from platforms such as the China Internet Joint Rumor Debunking Platform, Science Rumor Debunking, Popular Science China, and Kedou Wuxianpu, covering debunked information from August 2019 to December 2023. After applying the same processing methods to the real information, 3605 pieces are retained.

### 4.4. Experimental Analysis

#### 4.4.1. Comparison of Singal Models

This study compares several single models using a self-built dataset to evaluate the superiority of the proposed MIBKA-CNN-BiLSTM model for detecting false information. The single models included CNN, RNN, GRU, LSTM, and BiLSTM. The comparison of experimental results is shown in Table 7. All experiments are conducted under consistent operating conditions and parameter settings to ensure the reliability and reproducibility of the results.

BiLSTM achieves the best performance among all single models, with an 80.28% accuracy and the highest precision, recall, and F1-score. Compared to LSTM, BiLSTM improves accuracy by 1.57%, primarily due to its bidirectional structure. This design allows it to capture both forward and backward contextual information within the text sequence. This ability is crucial for identifying fake information, particularly when it involves implicit logic or misleading context. GRU performs slightly worse than LSTM but better than RNN, suggesting that its gated mechanism enhanced sequence modeling. RNN exhibits the weakest performance, with an accuracy of only 74.07%. This lower accuracy is mainly due to its susceptibility to gradient vanishing and limited capability in modeling long-term dependencies in text. CNN achieves an accuracy of 75.60%, outperforming RNN but falling behind LSTM and BiLSTM. This result suggests that CNN is relatively effective in extracting local semantic features and is particularly skilled at recognizing patterns at the phrase level. However, due to its limited structure, CNN struggled to model long-distance dependencies, resulting in performance degradation when handling longer and structurally complex text.

While CNN is limited when used independently, it is essential for extracting local features. Integrating it with BiLSTM is beneficial due to the complementary strengths of both models. CNN excels at identifying important phrases and structures in static text, while BiLSTM is designed to comprehend the sequence and context of words within the text. Although CNN may not be the highest-performing single model, it plays a crucial role in the hybrid structure by addressing BiLSTM’s limitations in local feature modeling and enhancing the overall discriminative capability of the model.

#### 4.4.2. Comparison of Hybrid Models

Several hybrid models based on single architectures are constructed using fusion optimization algorithms to verify the performance improvement effects of structural enhancement and parameter optimization. The hybrid models include CNN-BiLSTM, GWO-CNN-BiLSTM, WOA-CNN-BiLSTM, BWO-CNN-BiLSTM, and BKA-CNN-BiLSTM. A comparison of the experimental results is shown in Table 8. All the experimental conditions and settings are the same as the single modal comparison experiment.

Compared to the BiLSTM structure alone, the basic hybrid model CNN-BiLSTM has improved the accuracy by 1.55%, and all other metrics have also shown improvement. This result highlights the complementary nature of the CNN and BiLSTM structures. The CNN enhances the local perception ability of the input sequence, while the BiLSTM effectively models the semantic flow between contexts. Together, they provide a more comprehensive understanding of semantics.

The model’s performance improves significantly when the swarm intelligence optimization algorithm is used for adaptive hyperparameter adjustments in the CNN-BiLSTM model. The accuracy rates for the GWO-CNN-BiLSTM, WOA-CNN-BiLSTM, BWO-CNN-BiLSTM, and BKA-CNN-BiLSTM models exceed 83%, showing a marked improvement compared to the unoptimized CNN-BiLSTM. The BKA-CNN-BiLSTM achieves an accuracy of 84.94%, showcasing its robust global search capabilities and effective parameter configuration. Among all the models evaluated, the proposed model performs the best, with an accuracy rate of 88.05% and an F1-score of 86.71%. This improvement can be attributed to several factors. The circle mapping introduced by MIBKA during the population initialization stage enhances diversity. Additionally, a differential mutation mechanism improves the population’s exploration capabilities. Moreover, the logarithmic spiral reverse learning strategy is used in the position update process to prevent the model from getting trapped in local optima. Integrating these three strategies enables the model to maintain stability while finding the optimal hyperparameter combination, substantially enhancing overall detection performance.

#### 4.4.3. Comparison with Baseline Models

To better assess the model’s generalization ability across different datasets, we conduct comparative experiments using the Weibo21 dataset. Developed by Nan et al. in 2021, Weibo21 is a publicly available dataset for detecting fake information across various domains. It contains 4488 fake news items and 4640 real news items, all labeled with domain tags from nine distinct domains. We choose six baseline models to compare with the proposed model. The experimental results are presented in Table 9.

GRU-Attention [53]: This model uses a standard GRU with an attention mechanism to highlight important timesteps. However, despite incorporating the attention module, the GRU’s limited capacity and shallow structure hinder its ability to capture long-range dependencies. This limitation reduces its performance in complex reasoning tasks like fake information detection.

HSA-BiLSTM 54]: This model combines BiLSTM with a hierarchical self-attention mechanism to improve text structure and semantics modeling. Although it effectively extracts layered semantics, it struggles to capture complex semantic relationships and multi-paragraph content that may be misleading or fake. This limitation arises from its sensitivity to training data and a lack of specific tuning for the task at hand.

BERT-BiGRU [55]: This model mixes BERT’s deep contextual embeddings with BiGRU for sequential modeling. It benefits from BERT’s pretraining and BiGRU’s context modeling. However, GRU’s limited expressive power in handling long-text or deep-reasoning scenarios still leads to occasional misclassifications.

DE-BiLSTM [56]: This model uses DE to optimize the hyperparameters of the BiLSTM network. DE effectively addresses the challenge of local optima that often arises in a grid search, enabling a better capture of semantic and sequential features. However, DE’s performance is still limited by its initialization sensitivity and reduced efficiency in high-dimensional spaces.

BERT-CNN [57]: A lightweight architecture that integrates BERT and CNN to harness their strengths effectively. BERT provides rich semantic embeddings, while CNN captures local phrase-level features. Although this combination improves micro-level pattern recognition, the limited receptive field of CNN restricts its ability to model global dependencies. This limitation can affect performance in cases involving contextual jumps or logical confusion.

RoBERTa-WOA-CNN 58]: The model utilizes RoBERTa’s deep language modeling capabilities and incorporates the Whale Optimization Algorithm (WOA) for global hyperparameter tuning. Moreover, CNN layers are used for classification. Despite improvements in robustness and generalization, the model’s performance is still limited by WOA’s slower convergence and the CNN’s restricted ability to represent semantics in cross-domain scenarios.

MIBKA-CNN-BiLSTM: This model integrates CNN for local feature extraction, BiLSTM for modeling bidirectional dependency, and an improved BKA for hyperparameter optimization. As a result, it achieves precise tuning and optimal parameter combinations. Its lightweight structure ensures computational efficiency while maintaining high detection accuracy across domains.

The experimental results show that the model proposed in this paper outperforms all the baseline models every performance metric. Compared with the average of the six baseline models, MIBKA-CNN-BiLSTM improved the accuracy by 1.52% and the F1-score by 1.71%.

#### 4.4.4. t-SNE Analysis

**Figure 5:**
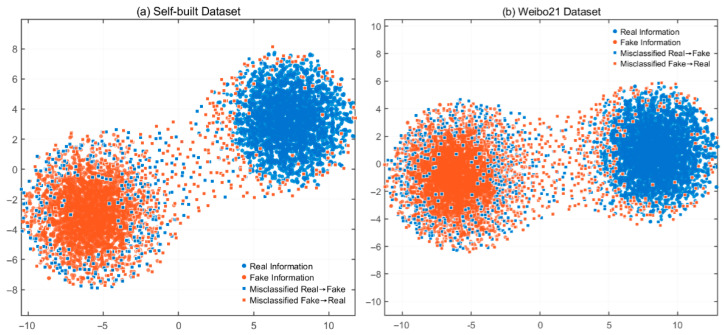
t-SNE visualization results.

t-Distributed Stochastic Neighbor Embedding (t-SNE) is a powerful nonlinear dimensionality reduction technique that effectively maps high-dimensional data into a two-dimensional space. This mapping helps intuitively understand the distribution of the data. In this study, t-SNE is employed to visualize and analyze the detection performance of the proposed model on both the self-constructed dataset and the public Weibo21 dataset. The results are shown in Figure 5.

In the self-constructed dataset, there are 2817 samples of real information and 3605 samples of fake information, where the model achieves an accuracy of 88.05%. The t-SNE plot clearly shows a significant separation between the samples of real and fake information in the two-dimensional space. This separation strongly indicates that the model successfully identifies the distinguishing features of both categories, enabling reliable classification. Nonetheless, some misclassified points scatter between the clusters of blue and orange dots. These misclassifications primarily result from two factors: the inherent ambiguity in the features of specific samples complicating the model’s judgment and the model’s partial failure to learn some fine-grained distinctions, leading to errors in classification. Meanwhile, the blue and orange dots display a certain degree of intra-class aggregation, clearly reflecting the high similarity among samples within the same category in the feature space. However, the presence of overlapping regions between the two types of data points further highlights that, in these regions, real and fake information share highly similar features, thus increasing the classification difficulty.

The Weibo21 dataset contains 4488 fake and 4640 real information samples. The model achieves an accuracy of 86.72%. The t-SNE visualization shows a separation trend between real and fake samples. However, the mixing degree between the two categories is more prominent than in the self-built dataset. This observation suggests that the Weibo21 dataset presents more complex data characteristics and blurrier class boundaries. Many misclassified points are scattered across the space, confirming the increased classification difficulty of this dataset. Numerous interfering factors and intricate feature relations hinder the model’s ability to distinguish between real and fake content accurately. Although both categories exhibit localized aggregation in certain regions, the level of clustering is lower than that observed in the self-constructed dataset. The significant overlap between the two classes indicates that the feature differences in Weibo21 are less distinct. Therefore, the model must learn more discriminative feature representations to improve classification performance.

A comparative analysis of both datasets reveals that the model performs better on the self-constructed dataset than on Weibo21. This improvement is primarily due to the feature distribution of the self-constructed dataset aligning more closely with the model’s assumptions and learning capabilities. In contrast, the complexity of the Weibo21 dataset exceeds some of the model’s expectations. Future work will optimize the model architecture to strengthen its ability to extract intricate features and enhance its performance on more complex datasets. Alternatively, introducing advanced feature engineering techniques can help identify more distinctive features, which may reduce misclassifications and significantly improve the model’s generalization capability.

## 5. Conclusions and Future Work

### 5.1. Conclusions

This paper proposes an improved MIBKA-CNN-BiLSTM hybrid model for detecting fake information. The model enhances the Black Kite Optimization Algorithm by implementing a triple strategy and optimizes the dual-channel deep learning architecture. A high-quality dataset has also been constructed and validated, providing a reliable foundation for model performance evaluation.

Firstly, the MIBKA achieves breakthrough improvements through three key strategies. The first strategy involves the reconstruction of population initialization using circle chaotic mapping, effectively addressing the uneven distribution caused by traditional random initialization. This approach significantly accelerates convergence on the CEC2017 benchmark tests. The second strategy introduces a random-to-elite differential mutation (DE/rand-to-best/1), which replaces the original BKA attack phase’s random perturbation mechanism, establishing a dynamic balance between global exploration and local exploitation. Experiments demonstrate its effectiveness in avoiding local optima in complex optimization tasks. The third strategy designs an LSOBL mechanism that guides the active exploration of the reverse solution space through spiral phase angles and dynamic boundaries, substantially improving search efficiency and robustness. These improvements collectively endow MIBKA with superior parameter optimization capabilities.

Secondly, this study constructs an efficient CNN-BiLSTM dual-channel feature extraction network and employs MIBKA for intelligent hyperparameter optimization. The CNN branch utilizes multi-scale convolutional kernels to precisely capture local anomalous patterns in text, such as modifier stacking and abnormal referencing. Meanwhile, the BiLSTM branch models long-range contextual logical dependencies, such as causal breaks and concept shifts, through bidirectional state propagation. The MIBKA jointly optimizes key hybrid parameters, including the number of convolutional kernels and LSTM units, enabling the model structure to meet task requirements adaptively. Experiments on the self-built dataset show that the optimized model achieves an accuracy of 88.05%, improving by 6.22% over the unoptimized baseline CNN-BiLSTM and by 3.11% compared to the best-performing alternative, validating the effective synergy of architecture and optimization strategies.

Moreover, the proposed model compares with other baseline models on the publicly available Weibo21 dataset, achieving an accuracy of 86.72%, which is 0.41% higher than the best baseline model. The result indicates that the proposed model performs exceptionally well on the self-built dataset and exhibits strong generalization capability on a cross-domain, multi-source heterogeneous public dataset. The benefit primarily arises from MIBKA’s integration of global search and local adaptive mechanisms within complex parameter spaces. This integration enables the CNN-BiLSTM framework to maintain significant expressive power and robust classification across various textual styles and semantic differences. t-SNE analyses further illustrate clear class separability on both datasets, providing solid empirical support for the experimental results.

In summary, this research thoroughly investigates the synergistic application value of swarm intelligence optimization algorithms and deep learning models in fake information detection. Experimental results demonstrate that MIBKA-CNN-BiLSTM significantly outperforms mainstream single models, hybrid models, and pre-trained models on both the self-built and Weibo21 datasets, highlighting its comprehensive advantages in feature extraction, parameter optimization, and cross-scenario generalization. The constructed dataset provides excellent resources for domain research, significantly enhancing the rigor and reliability of model validation.

### 5.2. Suggestion

The proposed MIBKA-CNN-BiLSTM model demonstrates significant practical application potential in detecting and preventing false information. The efficient, accurate, and adaptable model enables effective performance in real-world scenarios. One application is real-time content review. Due to its lightweight architecture, the model can be integrated into the social media platform’s backend system to scan posts, comments, and shared content in real time. As a result, the system can quickly mark potential false information and shorten its diffusion window period. Another application is trend prediction for dissemination. By combining with social network analysis, the model can identify new clusters of false information. The ability of BiLSTM to capture semantic evolution can predict the changing trends of false information during the dissemination process. This capability enables the platform to limit the spread of high-risk topics actively.

### 5.3. Limitations and Future Work

Although MIBKA-CNN-BiLSTM excels at detecting fake information, it still has limitations and areas that require improvement. First, the model’s ability to discern ambiguous features or complex texts is inadequate due to its architecture’s lack of depth in understanding deep semantic contradictions and covert narrative strategies. Future research could explore incorporating external knowledge graphs or fact verification modules to enhance the model’s recognition of implicit logical fallacies. Second, although the model exhibits better generalization than baseline models in cross-domain scenarios, there remains room for improvement. Future work should investigate domain adaptation techniques to mitigate performance degradation caused by data distribution shifts. Finally, this study focuses on textual modality. Future work could expand to multimodal fake information detection, exploring cross-modal consistency modeling and joint optimization frameworks.

## Figures and Tables

**Table 1:** Hybrid Models in Fake Information Detection.

Model	Key Features	Advantages	Limitations
CNN-RNN	local features + temporal dependencies	Fuses local-global features; suited for fake news	RNN weak in long-distance dependency modeling
CNN-BiLSTM	local features + bidirectional temporal modeling	Captures bidirectional context; robust to semantics	Performance relies on hyperparameters
CNN-BiGRU-Att	local features + simplified gating + attention mechanism	Faster convergence; attention focuses on key semantic regions, boosting detection accuracy	Attention adds computational overhead; may over-focus on noisy features
GNN	weight-adjusted + differentiable pooling	Integrates user factors; enhances graph aggregation	Relies on quality of user preference data
GCN/GNN/GAT	diverse graph models + pooling	Explores varied graph structures; improves down sampling	Performance varies by graph model

**Table 2:** Classification of chaotic maps.

Type	Included Maps	Mathematical Characteristics
Continuous	Circle, Sine, Logistic, Gauss, Sinusoidal, Cubic, Henon, Fuch, Chebyshev, ICMIC, Singer, SPM	Generate smooth, ergodic sequences via continuous nonlinear equations, suitable for continuous parameters
Discrete-segment	Bernoulli, Piecewise	Generate jump sequences via piecewise functions, suitable for discrete parameters
Hybrid-modal	Logistic-Tent-Cosine, Tent-Logistic-Cosine, Logistic-Sine-Cosine, Sine-Tent-Cosine, Iterative, Tent, Kent	Combine continuous and discrete features, adaptable to mixed parameter spaces

**Table 3:** Classification of mutation strategies.

Category	Mutation Strategy	Core Principle
Differential Evolution	DE/rand-to-best/1, DE/best/1, DE/rand/2, DE/best/2	Generate new solutions via difference vectors between random and elite individuals
Probability-Driven	Guass1, Guass2, Cauchy1, Cauchy2, H	Use stochastic perturbations from Gaussian or Cauchy distributions to adjust step size
Adaptive Regulation	t, Self-t, Periodic	Dynamically adjust mutation intensity according to iteration progress
Hybrid Heuristics	Cloud	Combine fuzzy logic with stochastic cloud models for uncertain perturbation

**Table 4:** Classification of OBL strategies.

Category	Strategies	Core Characteristics
Static Mapping	Basic OBL (OBL), Improved OBL (IOBL)	Generate opposite solutions using fixed formulas with constant boundaries
Adaptive Regulation	Adaptive OBL (AOBL), Dynamic OBL (DOBL)	Adjust opposite boundaries or perturbation strength dynamically based on iteration progress
Hybrid Heuristics	Chaotic OBL (COBL), Spiral OBL (SOBL) Logarithmic Spiral OBL (LSOBL)	Use nonlinear mechanisms like chaos and spiral functions to enhance diversity
Elite-Driven	Elite OBL (EOBL), Quasi OBL (QOBL), Weighted OBL (WOBL)	Utilize elite or best-performing individuals to guide opposite solution generation
Probability-Based	Beta OBL (BOBL), Fast Random OBL (FROBL)	Generate opposite solutions using Beta distribution or random sampling to balance exploration and exploitation

**Table 5:** Experimental parameter settings.

Module/Process	Parameter Name	Value/Range	Type	Description
MIBKA	Population Size N	50	Fixed	Number of individuals in the initial population
	Maximum Iterations $T_{max}$	100	Fixed	Stopping criterion for hyperparameter tuning
	Scaling Factor F	0.8	Fixed	Mutation factor in DE/rand-to-best/1
	Spiral Tightness k	0.2	Fixed	Controls LSOBL search scope
CNN-BiLSTM	Input Sequence Length	256	Fixed	Texts padded/truncated to uniform length
	Word Embedding Dimension	300	Fixed	GloVe pre-trained embedding dimension
	Number of Conv Layers $L_{conv}$	{1, 2, 3}	Discrete	Integer range for layer depth
	Number of Kernels K	{32, 64, 128}	Discrete	Number of kernels per convolutional layer
	BiLSTM Hidden Units $H_{lstm}$	{64, 128, 256}	Discrete	Number of hidden units in BiLSTM
	Dropout Rate β	[0.2, 0.6]	Continuous	Uniform sampling from interval
Training & Testing	Optimizer	Adam	Fixed	Learning rate set to 0.0001
	L2 Regularization λ	[1 × 10^−5^, 1 × 10^−3^]	Continuous	Log-uniform sampling
	Batch Size B	{32, 64, 128}	Discrete	Number of samples per batch
	Epochs	100	Fixed	Early stopping enabled (patience = 10)

**Table 6:** Confusion matrix for fake information detection.

Prediction/Actual	Real	Fake
Real	True Real (TR)	False Real (FR)
Fake	False Fake (FF)	True Fake (TF)

**Table 7:** Experimental results of the single models.

Model	Accuracy (%)	Precision (%)	Recall (%)	F1-Score (%)
CNN	75.60	74.55	71.19	72.81
RNN	74.07	72.77	69.49	71.10
GRU	77.15	76.33	72.88	74.58
LSTM	78.71	78.10	74.58	76.29
BiLSTM	80.28	79.87	76.27	78.07

**Table 8:** Experimental results of the hybrid models.

Model	Accuracy (%)	Precision (%)	Recall (%)	F1-Score (%)
CNN-BiLSTM	81.83	81.65	77.97	79.69
GWO-CNN-BiLSTM	84.36	83.92	81.08	82.48
WOA-CNN-BiLSTM	84.16	84.49	80.41	82.38
BWO-CNN-BiLSTM	83.41	83.41	79.66	81.51
BKA-CNN-BiLSTM	84.94	85.21	81.36	83.21
MIBKA-CNN-BiLSTM	88.05	88.74	84.75	86.71

**Table 9:** Baseline models comparison on the Weibo21 dataset.

Model	Accuracy (%)	Precision (%)	Recall (%)	F1-Score (%)
GRU-Attention [53]	83.64	84.07	82.88	83.47
HAS-BiLSTM [54]	84.32	84.11	83.93	84.02
BERT-BiGRU [55]	85.27	85.19	85.03	85.11
DE-BiLSTM [56]	85.74	85.89	85.33	85.61
BERT-CNN [57]	85.91	85.76	85.63	85.69
RoBERTa-WOA-CNN [58]	86.31	86.18	86.02	86.10
MIBKA-CNN-BiLSTM	86.72	86.89	86.54	86.71

## Data Availability

The original code and data presented in the study are openly available in GitHub at https://github.com/Kcoroo/MIBKA (accessed on 7 July 2025).
